# Supplementary material for: LLIN Evaluation in Uganda Project (LLINEUP) – Plasmodium infection prevalence and genotypic markers of insecticide resistance in Anopheles vectors from 48 districts of Uganda
Source: medRxiv. 2023 Aug 5:2023.07.31.23293323. Preprint. [Version 1] doi: 10.1101/2023.07.31.23293323 (PMC10418296; doi:10.1101/2023.07.31.23293323)
Supplement: Supplement 1 [file NIHPP2023.07.31.23293323v1-supplement-1.pdf]

Supplementary table 1: Prevalence of *Plasmodium* positive mosquitoes collected from 104 health sub districts across all 5 collection rounds. [1] Data for *Plasmodium falciparum* and for *P. ovale*, *P. vivax* and *P. malariae* combined. [2]Data from only 90 HSDs due to COVID-19 impacts.

	**Plasmodium[1] species**	**Round 1 (Baseline)**	**Round 2**	**Round 3**	**Round 4**	**Round 5[2]**
An. gambiae s.s.	*P. falciparum*	5.6% (n=1284)	4.2% (n=191)	1.4% (n=441)	3.5% (n=256)	4.0% (n=815)
	P. OVM	1.2% (n=1284)	0.5% (n=191)	0.4% (n=441)	0.4% (n=256)	0.5% (n=815)
***An.* arabiensis**	*P. falciparum*	0% (n=80)	0% (n=36)	0% (n=61)	0% (n=117)	0% (n=74)
	P. OVM	0% (n=80)	0% (n=36)	0% (n=61)	0% (n=117)	0% (n=74)
An. funestus	*P. falciparum*	3.5% (n=432)	2.6% (n=194)	2.4% (n=250)	2.1% (n=719)	3.9% (n=435)
	P. OVM	1.4% (n=432)	0.5% (n=194)	1.6% (n=250)	0.0% (n=719)	0.7% (n=435)

Supplementary figure 1: Infection prevalence of *P. falciparum* and *P.ovale + vivax + malariae (ovm)* in *An. gambiae* stratified by resistance associated marker genotype. There was no significant association between infection with either parasite grouping and genotype.

Vgsc-995

genotype	N	P. falciparum	P. ovm
LL	3	0 (0%)	0 (0%)
LS	19	2 (10.5%)	0 (0%)
FL	1	0 (0%)	0 (0%)
FS	383	18 (4.7%)	4 (1%)
SS	2484	102 (4.1%)	19 (0.8%)
FF	50	2 (4%)	1 (2%)
	Fisher test P =	0.47	0.41

Cyp6p4-236M

genotype	N	P. falciparum	P. ovm
II	35	0 (0%)	0 (0%)
IM	493	20 (4.1%)	5 (1%)
MM	2398	103 (4.3%)	19 (0.8%)
	Fisher test P =	0.63	0.69

Coeae1d

genotype	N	P. falciparum	P. ovm
SS	647	26 (4%)	5 (0.8%)
SR	1435	63 (4.4%)	10 (0.7%)
RR	854	35 (4.1%)	9 (1.1%)
	Fisher test P =	0.94	0.62

Cyp4j5-43F

genotype	N	P. falciparum	P. ovm
LL	557	20 (3.6%)	7 (1.3%)
LF	1088	49 (4.5%)	10 (0.9%)
FF	1264	53 (4.2%)	6 (0.5%)
	Fisher test P =	0.71	0.16

2La inversion

genotype	N	P. falciparum	P. ovm
++	1224	50 (4.1%)	9 (0.7%)
+a	997	47 (4.7%)	8 (0.8%)
aa	559	22 (3.9%)	7 (1.3%)
	Fisher test P =	0.71	0.53
